# Zebrafish as a Potential Model Organism for Drug Test Against Hepatitis C Virus

**DOI:** 10.1371/journal.pone.0022921

**Published:** 2011-08-08

**Authors:** Cun-Bao Ding, Jing-Pu Zhang, Ye Zhao, Zong-Gen Peng, Dan-Qing Song, Jian-Dong Jiang

**Affiliations:** 1 Institute of Medicinal Biotechnology, Chinese Academy of Medical Sciences and Peking Union Medical College, Beijing, China; 2 College of Chemical Engineering and Biotechnology, Hebei Polytechnic University, Tangshan, Hebei, People's Republic of China; 3 State Key Laboratory of Bioactive Substances and Functions of Natural Medicines, Institute of Materia Medica, Chinese Academy of Medical Sciences and Peking Union Medical College, Beijing, China; University of South Carolina School of Medicine, United States of America

## Abstract

Screening and evaluating anti- hepatitis C virus (HCV) drugs *in vivo* is difficult worldwide, mainly because of the lack of suitable small animal models. We investigate whether zebrafish could be a model organism for HCV replication. To achieve NS5B-dependent replication an HCV sub-replicon was designed and created with two vectors, one with HCV ns5b and fluorescent rfp genes, and the other containing HCV's 5′UTR, core, 3′UTR and fluorescent gfp genes. The vectors containing sub-replicons were co-injected into zebrafish zygotes. The sub-replicon amplified in liver showing a significant expression of HCV core RNA and protein. The sub-replicon amplification caused no abnormality in development and growth of zebrafish larvae, but induced gene expression change similar to that in human hepatocytes. As the amplified core fluorescence in live zebrafish was detectable microscopically, it rendered us an advantage to select those with replicating sub-replicon for drug experiments. Ribavirin and oxymatrine, two known anti-HCV drugs, inhibited sub-replicon amplification in this model showing reduced levels of HCV core RNA and protein. Technically, this method had a good reproducibility and is easy to operate. Thus, zebrafish might be a model organism to host HCV, and this zebrafish/HCV (sub-replicon) system could be an animal model for anti-HCV drug screening and evaluation.

## Introduction

Current therapy for HCV infection is the combination of Pegylated-interferon (PEG-IFN) with ribavirin [1], [2]. This regimen has only suboptimal clinical efficacy and is with side-effects [3], [4]. It is widely agreed that a suitable small animal model for HCV replication would significantly accelerate discovery of new drugs against HCV. Although investigation on mouse HCV models is progressing, the results were far from satisfactory [5]. Transgenic mice made with HCV genetic elements were created in order to identify HCV pathogenic components, and were short of HCV enzyme-controlled viral replication [6]–[9]. All of the HCV infection mouse models need surgical operation to inoculate human liver cells into recipient organs [8]. Difficulties of using the models include complicated surgical procedures, genetic variation of the clinical HCV isolates, low and/or unstable HCV infection rate, low HCV viraemia [10], [11], as well as the safety concerns that arise from the dangers of HCV transmission to research personnel via rodent bite during experiments. These obstacles have largely restricted the use in drug testing [11]. Novel, small and easy-to-handle model organism for *in vivo* HCV replication represent a pressing need for anti-HCV drug discovery.

HCV belongs to the Flaviviridae family and has a single-stranded positive RNA genome [12]. HCV genome encodes a single open reading frame (ORF) and is translated into a polyprotein, which is cleaved into four structural proteins (core, E1, E2 and p7) and six non-structural ones (NS2, NS3, NS4A, NS4B, NS5A and NS5B) [12]. Among these factors, NS5B is necessary for viral replication with essential assistance from 5′UTR (IRES) and 3′UTR [13], [14]. NS5B is a 65 kD viral RNA-dependent RNA polymerase (RDRP). It recognizes specific RNA 3′UTR sequence and initiates HCV replication for the negative strand RNA, which then works as a template to create positive strand of RNA for HCV assembly, therefore completing the amplification of HCV genetic materials [12]. During this process the 3′UTR is a crucial element for HCV replication [15]. Based on the functions of the above mentioned HCV elements, a HCV sub-genomic replicon (sub-replicon) was designed and created in our laboratory. In selecting host recipients for the HCV sub-replicon, zebrafish as a vertebrate came to our attention. Zebrafish has been used to the study of human diseases [16]–[18]. As zebrafish has a good homology to human in genetics, particularly in liver [18], and is small and easy-to-handle in laboratory, we explored its potential of being a model organism for HCV replication. In what follows we show that zebrafish could be a potential small animal model for replication of the HCV sub-replicon and used in drug evaluation.

## Materials and Methods

### Plasmids

**Figure 1:**
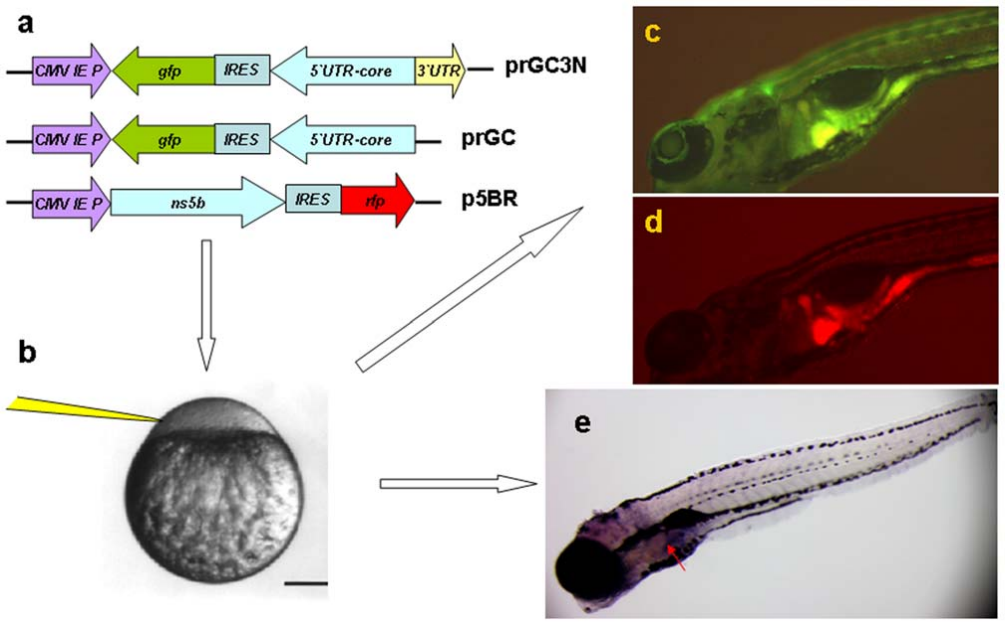
Zebrafish as a model organism for HCV sub-replicon amplification. a. The HCV sub-replicon was created with prGC3N and p5BR vectors. Cartoons are for vector prGC3N, prGC and p5BR. CMV promoter was for transcription. prGC3N has a gfp-IRES(EMCV)-core-5′UTR sequence that was reversely inserted at the downstream of CMV promoter and followed by a HCV 3′UTR sequence in a forward direction; prGC has no HCV 3′UTR; the p5BR is a functional vector carrying HCV RNA polymerase (NS5B) and RFP. b. Co-injection of the sub-replicon into zebrafish zygote blastomere. c. Fluorescent microscopy examination for HCV core protein amplification in zebrafish liver using a GFP filter (480 nm excitation, 505 nm emission; image, 100X). d. Fluorescent RFP filter (556 nm excitation, 586 nm emission) was used to detect liver HCV NS5B protein signal in red. e. The whole mount in situ nucleic acid hybridization was used to detect the positive strand of HCV core RNA, in order to confirm the green fluorescent signal for the replication of the HCV sub-replicon.

HCV 1b (J4L6s) strain (accession no. AF054247) was from Dr. HS Chen (Peking Union Medical College, Beijing, China). NS5B construct p5BR were made via insertion of the whole *ns5b* cds at the downstream of the CMV promoter in the pIRES2-DsRed (Fig. 1a). The prGC3N was constructed with the negative sequence of EGFP-IRES (form pIRES2-EGFP) and HCV core-5′UTR in a reverse-direction, and then, a positive strand HCV 3′UTR was attached to the 5′UTR in a forward-direction (Fig. 1a). The construct of prGC null of HCV 3̀UTR (Fig. 1a) served as a control. All of the constructs were confirmed by DNA sequencing.

### Zebrafish microinjection and fluorescent microscopic examination

Adult zebrafish (*Danio rerio*) AB line were obtained from Dr. Anming Meng (Tsinghua University, Beijing, China) and received human care and that study protocols comply with the institutions guidelines. Zebrafish were maintained in a controlled environment with 14-h light/10-h dark cycle at 28±1°C. The HCV sub-replicon zebrafish was made with co-injection of the prGC3N and p5BR into the blastomere at the early 1–8 cell-stage embryos at a concentration of 1 ng/µl of the construct DNA. Larvae positive for both GFP and RFP fluorescence were examined at 8 and 12 days post fertilization (dpf), using fluorescence microscopy with the GFP (480 nm excitation, 505 nm emission) and RFP filters (556 nm excitation, 586 nm emission), respectively.

### Reverse transcription - polymerase chain reaction (RT-PCR)

Larvae total RNA was isolated with Trizol Reagent. The cDNA was synthesized from 1 µg of the total RNA using AMV reverse transcriptase (Promega). The primers used were given in Table 1. The target cDNA templates were amplified by PCR with Taq polymerase (TaKaRa, Japan). PCR was performed with 0.5 µl cDNA and primer pairs of core, ns5b and β-actin, using a program of 94°C for 5 min, 94°C for 60 s, 55°C for 30 s and 72°C for 1 min. After 30 cycles, the reaction mixtures were incubated at 72°C for an additional 10 min to allow a complete synthesis. The RT-PCR products were subjected to 1.5% agarose gel electrophoresis. β-actin was used as control.

**Table 1:** Primer sequences for PCR reaction.

Primer	Sequences
Core F	5′-AGCGGTCGCAACCTCGTGGAA-3′
Core R	5′-GCGGAAGCTGGGATGGTCAAAC-3′
NS5B F	5′-GCTCGCCTTATCGTATTCC-3′
NS5B R	5′-AGTCGTCAGCACGCCAC-3′
Gfp F	5′-ACGGCGTGCAGTGCTT-3′
Gfp R	5′-TGGGTGCTCAGGTAGTGG-3′
3UTR-R	5′-ACATGATCTGCAGAGAGGCCAGTAT-3′
β-actin-F	5′-AGGGAAATCGTGGGTGACATCAAA-3′
β-actin-R	5′-ACTCATCGTACTCCTGCTTGCTGA-3′
Chemokine20F	5′-TCTCTTCTCACCTGCCCTAA-3′
Chemokine20R	5′-ATTGCTTGCACCTTCTCCCTC-3′
AHSG F	5′-GGAAGGCAGCGGTGAAA-3′
AHSG R	5′-ATGGTCTGGCCCGAGTG-3′
Hsp70 F	5′-GCGACACCTCTGGAAAC-3′
Hsp70 R	5′-TGCTCAGCCTGCCCTTG-3′
Rasgbd F	5′-ATCCCTCAACTTCCCACC-3′
Rasgbd R	5′-TCTGCCTGCTCCACCTC-3′
Argsyn F	5′-GACAGGACGAGGACTTTG-3′
Argsyn R	5′-TGACGGGAACAGGAATG-3′
ScarF2 F	5′-CTCTTGCGTCTACAGGG-3′
ScarF2 R	5′-GCTCAGCGGTTTCTATT-3′
Leugpcr F	5′-GGTGTTTGTCTGGGTTG-3′
Leugpcr R	5′-GGTCTGAGTGAAGAGGGA-3′
β-actin qF	5̀-TCTGGTGATGGTGTGACCCA-3̀
β-actin qR	5̀-GGTGAAGCTGTAGCCACGCT-3̀
Core qF	5̀-GCGACAACCTATCCCAAAG-3̀
Core qR	5̀-CCCAAGTTACGCGACCTAC-3̀

### Quantitative real time RT-PCR

The study RNA was reversely transcribed into cDNAs first. Then, the cDNA templates were used for quantitative PCR reaction. It was performed using the SYBR green technique in the Bio-Rad CFX96 system (Bio-Rad, Hercules, CA). Core and β-actin cDNA were detected with Platinum SYBR Green qPCR SuperMix-UDG kit (Invitrogen). Primers were designed with Primer 5.0 (Premier Co., Canada). The primers for core and β-actin cDNA detection included β-actin qF, β-actin qR and core qF, core qR (Table 1). Thermocycling conditions were the following, 5 min at 95°C, then 40 three-temperature reaction cycles with 30 s at 95°C, 30 s at 55°C and 30 s at 72°C. The data were analyzed using Bio-Rad CFX96 software. The copy number of the study genes in the untreated zebrafish larvae was defined as 1, and the numbers of gene copies in the treated larvae were plotted relative to that value.

### Western blotting

Zebrafish larvae protein was extracted with lysis buffer and separated in the 12% SDS-PAGE. The protein bands were transferred onto a nitrocellulose membrane followed by blocking. The membranes were incubated with anti-core or anti-NS5B antibodies (Abcam Co.) at 1∶2000 dilutions in TBS containing 1% skim milk; then the membrane was washed and incubated with HRP-conjugated goat anti-mouse or goat anti-rabbit IgGs (1∶2000 dilutions, Zhongshanjinqiao Co.) for 1 hr at RT. Proteins were detected using the Supersignal ® West Pico chemiluminescent substrate (Thermo) with AlphaEase® FC Imaging System (Alpha Innotech Corporation).

### Whole mount in situ hybridization

Core (nt 430–702) and ns5b (nt 8067–8459) sequence of the J4L6 strain were used as templates for hybridization probe synthesis, using DIG RNA Labeling Kit (Roche Diagnostics Scandinavia AB, Bromma, Sweden). Whole mount *in situ* hybridization was performed as previously described [19], [20]. Briefly, 10-dpf larvae were fixed with 4% paraformaldehyde for 10 hrs at 4°C and washed with 1×PBST. The larvae were treated with proteinase K and DNase I separately, pre-hybridized in 65°C for 4 hrs, and hybridized with the RNA probe at 65°C overnight. The residual probe was washed with 0.2×SSC, followed by incubating with anti-Dig-AP (Roche) at 4°C over night. After wash with 1×PBST, the samples were colorized with BCIP/NBT for 30 min and stopped with 1×PBST washing. The larvae were observed with light microscope.

### Drug treatment

Ribavirin and oxymatrine were from National Institutes for Food and Drug Control (Beijing, China). Ribavirin at a final concentration of 1000, 100 and 10 µg/ml, or oxymatrine at a final concentration of 200, 20 and 2 µg/ml was added into the zebrafish cultivation water, and incubated for 5 days (from 5 dpf to 10 dpf). Then, the larvae were collected for analysis.

### Ethics statement

This study was carried out in strict accordance with the recommendations in the Regulation for the Management of Laboratory Animals of the Ministry of Science and Technology of China. The protocol was approved by the Committee on the Ethics of Animal Experiments of the Institute of Medicinal Biotechnology, Chinese Academy of Medical Sciences (IMBF20060302).

## Results and Discussion

### Replication of fluorescence-labeled HCV sub-replicon in zebrafish larva

The fluorescence-labeled HCV sub-replicon was designed and constructed with a pair of vectors; one vector contained HCV core gene, 5′UTR sequence and fluorescent *gfp* in a reverse-direction to the CMV promoter, as well as a 3′UTR sequence in a forward-direction; and the other one contained fluorescent *rfp* and HCV ns5b genes (Fig. 1a). The two vectors in 1∶1 ratio were co-injected into zebrafish zygotes under microscope (Fig. 1b). By principle, the CMV promoter should initiate the transcription for the negative strand RNA of *gfp*-IRES-HCV/*Core*-5′UTR with a downstream sequence of the positive strand HCV 3′UTR RNA, which is for NS5B to recognize. Subsequently, the positive strand HCV *core* mRNA and *gfp* mRNA should be generated by NS5B (red signal) through a HCV 3′UTR-mediated amplification, and then translated into core and GFP proteins with a demonstration of green fluorescent signal. The green signal of GFP showing replication of the HCV sub-replicon (Fig. 1c) and red signal of RFP (Fig. 1d) for NS5B protein translation should be visible in live zebrafish under fluorescent microscope, if the sub-replicon amplifies in a sufficient amount. It allows us to select live zebrafish with active HCV sub-replicon amplification to enter into drug experiments. Amplification of the HCV sub-replicon in zebrafish liver could be confirmed by whole mount *in situ* nucleic acid hybridization which is used to test the presence of the positive strand HCV core mRNA (Fig. 1e).

**Figure 2:**
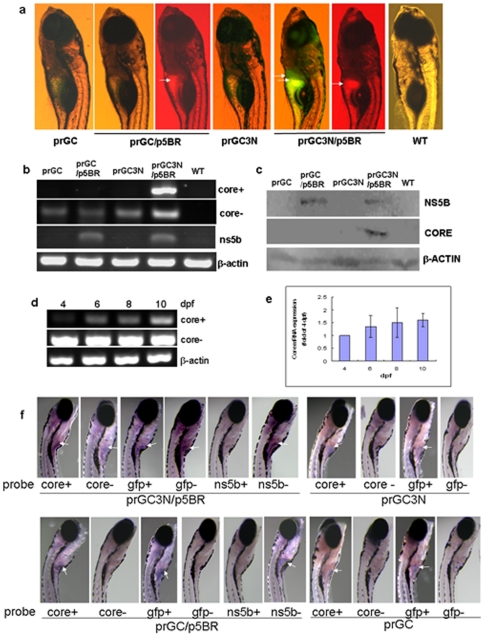
Amplification of the HCV sub-replicon in liver of the zebrafish larvae. a. HCV sub-replicon amplified in those co-injected with prGC3N and p5BR vectors. Green fluorescence represents the NS5B-dependent HCV core protein products of the sub-replicon (prGC3N/p5BR); red fluorescence indicates the presence of the NS5B enzyme. Arrows point the positive signals in liver area. WT, untreated control. b. RT-PCR measurement for the amplification of the study genes in the sub-replicon. Core+, the positive strand of HCV core RNA; core-, the negative strand of HCV core RNA; ns5b, HCV ns5b from p5BR; and beta-actin, loading control. WT, wild type. c. Western blot for HCV core and NS5B proteins with anti-core or anti-NS5B antibody, respectively. Beta-actin was loaded as a control. d. Amplification of the HCV sub-replicon exhibits a time–dependent increase from 4- to 10-dpf in zebrafish larvae for the positive strand of core RNA; negative strand core RNA and beta-actin remains to be constant. dpf, day post fertilization. e. Confirmation of the time–dependent amplification of the HCV sub-replicon with quantitative real time RT-PCR for the positive strand of HCV core RNA. f. Whole mount in situ hybridization of the co-injected zebrafish. Whole mount in situ hybridizations were carried out on 10-dpf larvae using antisense or sense RNA probes. The presented are original microscopy images of the zebrafish larvae (80X). dpf, day post fertilization.

The experimental results are shown in Fig. 2a. Eight days after co-injection of the prGC3N and p5BR vectors, both green and red fluorescence was detected in the liver location of zebrafish, indicating a 3′UTR-mediated amplification of the HCV sub-replicon. The successful rate of sub-replicon amplification was seen in about 30% of the zebrafish zygotes co-injected with prGC3N and p5BR. Control larva groups injected with prGC or prGC3N alone showed neither green nor red fluorescent signal; co-injection of prGC and p5BR which was null of the 3′UTR sequence exhibited red signal only. These results indicate that the green signal from the larvae co-injected with prGC3N and p5BR vectors represents the sub-replicon replication mediated through an interaction between HCV 3′UTR and NS5B. The experiment has been repeated over 7 times. The result suggests that this sub-replicon device encloses functional elements for HCV amplification in this model organism host.

To verify replication of the HCV sub-replicon in this model, HCV core mRNA and protein in the zebrafish larvae was isolated and examined with RT-PCR as well as Western blot. As shown in Fig. 2b, the negative strand HCV core RNA was detectable in all of the groups injected with prGC, or prGC3N, or prGC plus p5BR, or prGC3N plus p5BR, indicating an active transcription for the negative strand core RNA in these zebrafish larvae (Fig. 2b). However, the positive strand HCV core RNA was detected only in the zebrafish larvae co-injected with prGC3N and p5BR (prGC3N/p5BR group), but not in those injected with prGC or prGC3N alone, or p5BR and prGC in combination (prGC/p5BR) (Fig. 2b). The results were further confirmed at protein level using Western blot assay, showing the HCV CORE protein signal presented only in the prGC3N and p5BR co-injection group (Fig. 2c). To learn the time-dependent dynamics, amplification of the sub-replicon was followed up for 10 days after prGC3N/p5BR co-injection. As shown in Fig. 2d, the level of HCV core mRNA significantly increased along with time, indicating an active amplification process of the HCV sub-replicon in larvae liver. The amplification kinetics was validated with real time RT-PCR (Fig. 2e).

The green and red fluorescent signal localized mainly in the liver area of the larva body (Fig. 2a), indicating a liver-selective tendency of the HCV sub-replicon. To further verify the selectivity, *in situ* hybridization was done for the whole larva body 10 day post fertilization using core and egfp nucleic probes. The goal is to learn the *in vivo* distribution of the sub-replicon products. As shown in Fig. 2f, the positive strand HCV core RNA signal, probed with negative strand core sequence, was detected only in the larvae co-injected with prGC3N/p5BR vectors, and concentrated in the liver area of the larvae, providing a supportive result for that in Fig. 2a. As a matter of fact, in the prGC3N/p5BR group the signal detected with either positive and/or negative strand nucleic acid sequences for core or GFP presented predominantly in larva liver area, with very weak signal in intestine (Fig. 2a). Wild type larvae were without signal (Fig. 2a). The experiment was repeated 3 times. Detection of HCV core expression in zibrafish organs was not done in this study because the larvae at 10 days were about 4 mm in length, too small to precisely separate their organs.

### Biological impact on zebrafish larva after prGC3N/p5BR co-injection

**Figure 3:**
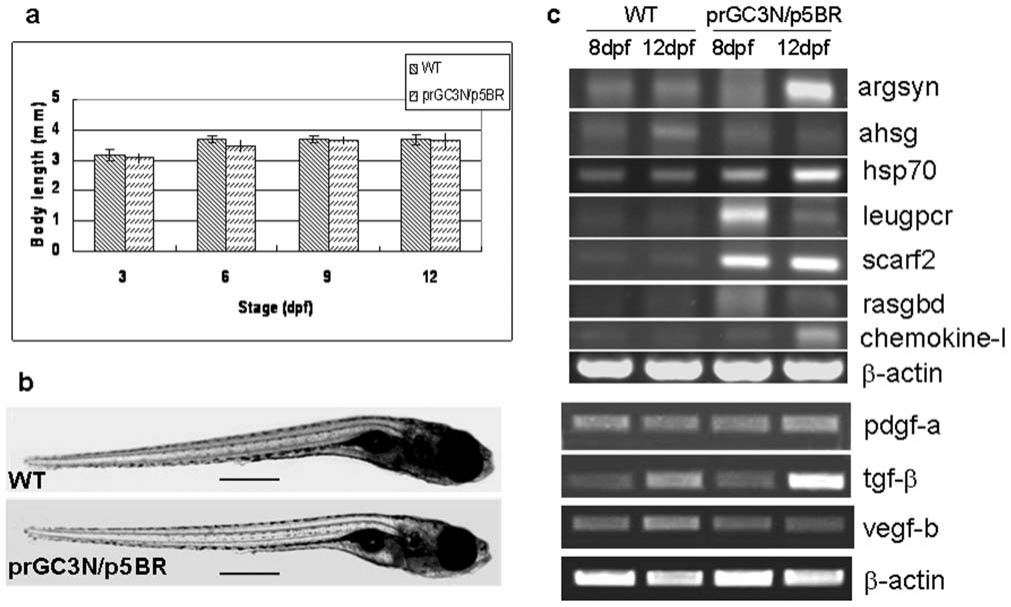
Biological effects on zebrafish larvae after injection of the HCV sub-replicon. a. Body length of the wild type larvae and those co-injected with prGC3N/p5BR. b. Phenotype of wild type larvae and those co-injected with prGC3N/p5BR. The scales represent 500 um. c. Expression of the HCV-associated genes in zebrafish larvae. The mRNA level of the study genes were examined with RT-PCR reaction in wild-type and the prGC3N/p5BR co-injection larvae (8 dpf and 12 dpf).

Body length and phenotype were compared between wild type larvae and those co-injected with prGC3N/p5BR. As shown in Fig. 3a, amplification of HCV sub-replicon did not slow down the growth of the larvae (*p*>0.05) at least in the first 12 days of embryonic development; deformity phenotypes were not observed as well (Fig. 3b). The results indicated that development and growth of the zebrafish at the stage from zygote to larva was not interrupted by the prGC3N/p5BR co-injection or the subsequent HCV sub-replicon amplification. The sub-replicon positive zebrafish survived throughout the larva stage (for at least 12 days).

HCV infection causes host gene expression alteration in human liver cells. The reported genes up-regulated by HCV infection includes chemokine-1 1igand (chemokine-l) [21], Solute carrier family 2 (ScarF2) [22], Leucine-rich repeat-containing G protein-coupled receptor 5 (Leugpcr) [23], Alpha2-HS glycoprotein (AHSG) [24], heat stress protein 70 (Hsp70) [25], Ras-related GTP binding D (Rasgbd) [23], Argininosuccinate synthetase 1 (Argsyn) [23], as well as profibrogenic molecules like TGF-β, PDGFs, and VEGF [26] et al. Thus, our next experiment is to learn whether, or not, replication of the sub-replicon in larvae causes such change in these genes. RT-PCR was done for the study genes. The results are shown in Fig. 3c. Eight and twelve days after co-injection of the prGC3N/p5BR vectors the expression of Argsyn, Hsp70, Leugpcr, ScarF2, Rasgbd and chemokine-1 genes in the larvae increased as compared to the untreated wildtype controls; but the AHSG gene appeared being down-regulated. For the profibrogenic molecules, TGF-β increased but the expression change of PDGFa and VEGFb was not obvious in this study. The results in zebrafish basically agreed with that in HCV infected human liver cells [23], [27], showing a similar transcription impact on the host. These results suggest that zebrafish liver might have an intracellular circumstance supportive for HCV replication and comparable to that in human hepatocytes.

### Anti-HCV drug evaluation using zebrafish as a model organism

**Figure 4:**
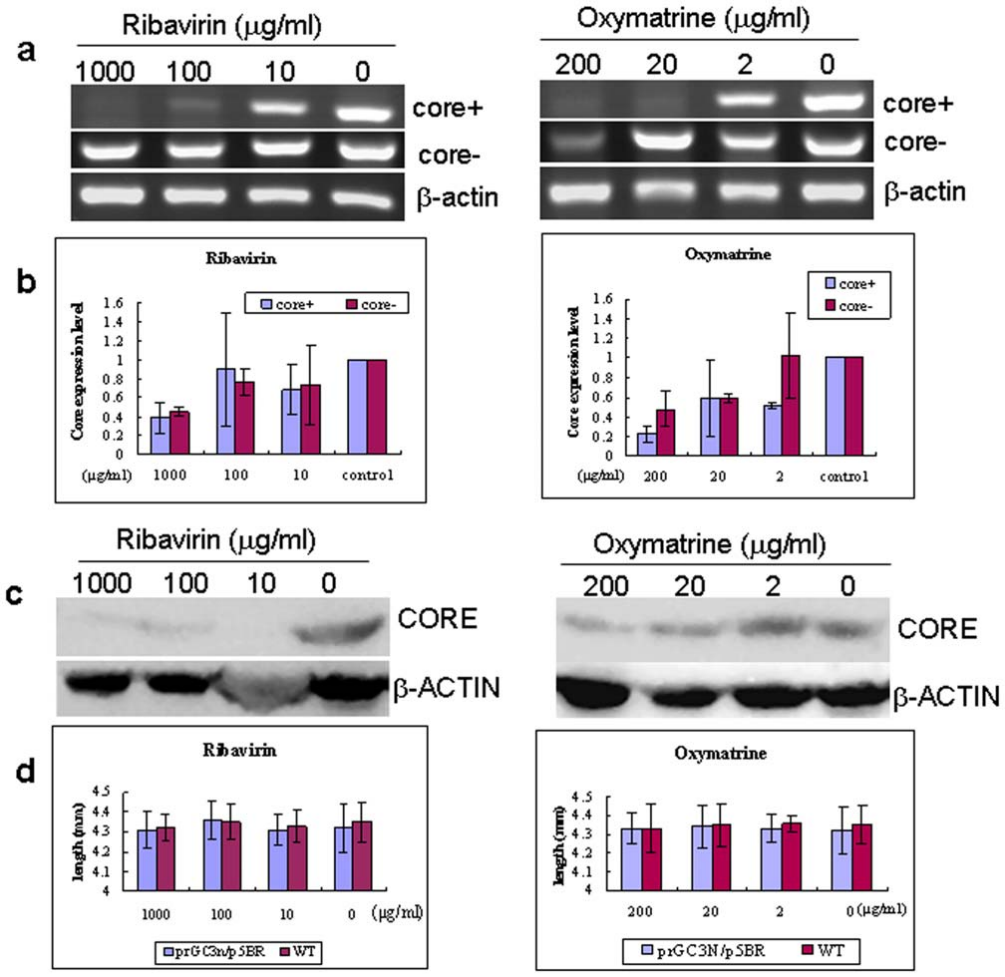
Ribavirin and oxymatrine inhibited amplification of the HCV sub-replicon in zebrafish larvae. Zebrafish larvae co-injected with prGC3N/p5BR were treated with ribavirin or oxymatrine respectively from 5-dpf to 10-dpf, followed by biological measurements. a. Conventional RT-PCR for anti-HCV effect of the study drug. Core+: the positive strand of core RNA; core-: negative strand of HCV core RNA. b. Real time RT-PCR examination for anti-HCV effect of the study drug. Core+: the positive strand of core RNA. c. Western blot assay for HCV core and NS5B proteins in the zebrafish larvae treated with ribavirin or oxymatrine. Core was detected with anti-HCV core antibody; beta-actin was with anti-beta-actin antibody (used as a control). d. Body length (mm) of the co-injected zebrafish larvae treated with the study drug (ribavirin or oxymatrine). Wild type larvae are shown as reference.

Next, we tested if the zebrafish HCV sub-replicon model was suitable for evaluation of anti-HCV drugs. The test compounds were ribavirin and oxymatrine. Ribavirin is currently the first-line clinical drug for HCV [28]. Oxymatrine is also an agent active against HCV replication in hepatitis C patients and has been used in Chinese hospitals [29]. Good water-solubility was another reason to select the two drugs. In this experiment, the drugs were added into the incubation water of the sub-replicon positive zebrafish larvae on day 5 of the embryonic development, with 20 zebrafish larvae in each treatment group. The experiment was followed up for 5 days. None of the larvae died during the treatment course, indicating a good safety of the drug at the study doses. The larvae were taken for total RNA extraction on day 5 post drug treatment. As shown in Fig. 4a, incubation of the larvae in the ribavirin- or oxymatrine-containing water for 5 days largely inhibited the amplification of the HCV core RNA indicated by positive strand of the sub-replicon. The inhibition was in a dose-dependent manner both in ribavirin and oxymatrine treated groups. Ribavirin at 100 µg/ml or oxymatrine at 20 µg/ml, or higher, significantly suppressed the replication of the HCV sub-replicon. The negative strand core RNA was exhibited as a reference in the figure in order to show a successful co-injection of the prGC3N/p5BR vectors into the zebrafish zygotes (Fig. 4a). Real time RT-PCR assay showed a similar result (Fig. 4b). The treatment efficacy of the drugs on HCV RNA was confirmed at protein level using Western blot (Fig. 4c). The results indicate that the zebrafish-hosted HCV sub-replicon amplification system appears to be a suitable animal model to evaluate anti-HCV drugs or drug candidates, especially for water-soluble agents. Fig. 4d demonstrates measurement of the larva body length at the end of the experiments and showed that neither ribavirin nor oxymatrine at the doses used caused reduction of the larva body length, verifying good safety of the drugs in clinic. Also, no deformity phenotypes were observed in the treated larvae.

The present study provides evidence that zebrafish liver cells might contain biological circumstances comparable to that of human hepatocytes. It could be one of the explanations of why zebrafish liver is permissive for human HCV amplification. Results from cancer study have drawn similar conclusion [18]. To the best of our knowledge this is the first report using zebrafish as an *in vivo* model organism to host HCV amplification and to evaluate anti-HCV drugs. In creating HCV sub-replicon inoculated zygotes, it is our experience that about 250 zebrafish zygotes could be injected per hour by a well-trained researcher, and the successful rate of injection is approximately 90%; among these zygotes about 30% turn out to be positive for the sub-replicon amplification. It enables us to obtain a good number of zebrafish larvae for experiments. As compared with mouse HCV models [5], [10], [30], [31], this model organism has at least the following advantages in drug test. First, the procedure of creating the sub-replicon positive larva is not complicated; second, the HCV RDRP-dependent sub-replicon replicates actively and steadily in zebrafish liver; third, the designed GFP fluorescent signal in live zebrafish for HCV core replication allow researchers to select those with active sub-replicon amplification to enter into drug experiments; and forth, this easy-to-handle small biological model seems to be suitable for drug screening, with no need to concern about the safety issues that arise from possible animal bite. The major disadvantages of this model are that 1) the HCV sub-replicon is eventually an artificial sub-virus and not the natural HCV, and 2) it is probably only suitable for water-soluble drugs and the dose calculation is not convenient. Currently, we are exploring the possibility of growing full genome HCV in zebrafish model.

Ribavirin and oxymatrine are known drugs for HCV in clinic [28], [29]. The anti-HCV efficacy of ribavirin is statistically significant [32], [33], although the molecular mechanism in its action against HCV remains to be clarified. Oxymatrine is an alkaloid extracted from the plant Kushen (Sophora flavescens Ait) [34]. Clinical studies in China has showed that injection of oxymatrine (0.4 g/d for 6 months) in chronic hepatitis B (CHB) patients caused over 40% of the patients to become negative for HBV DNA [35], and viral load measurement showed that oxymatrine injection (0.4 g/d for 3 months) decreased HBV viral load by about 2logs in average [36]. Further clinical investigations showed that oxymatrine (0.6 g/per, intramuscular injection for 3 months) also inhibits HCV replication in hepatitis C patients causing negative conversion of HCV RNA in about 30% of the patients [37]. In the present study, the two drugs were used as positive references to evaluate the zebrafish HCV model. Indeed, both drugs demonstrated an inhibiting effect on the replication of the HCV sub-replicons in this model organism at RNA as well as protein levels. The results agree with that observed in hepatitis C patients [33], [37]. In addition, the two drugs showed no toxicity to the larvae during the treatment, consistent with the safety record of the drugs in clinic [35], [38]. This drug experiment validates the usefulness of this model organism in anti-HCV drug evaluation. We consider the zebrafish HCV sub-replicon model a valuable tool with significant potential of being used to screen and evaluate anti-HCV drugs.
